# First line of defence: the role of sloughing in the regulation of cutaneous microbes in frogs

**DOI:** 10.1093/conphys/cou012

**Published:** 2014-04-21

**Authors:** Rebecca L. Cramp, Rebecca K. McPhee, Edward A. Meyer, Michel E. Ohmer, Craig E. Franklin

**Affiliations:** School of Biological Sciences, The University of Queensland, Brisbane, Australia

**Keywords:** Chytridiomycosis, global climate change, green tree frog, immune system, moulting, skin

## Abstract

Amphibian populations worldwide are currently experiencing unprecedented declines due to the combined effects of emerging infectious disease and climate change. The skin is the first line of defence in preventing establishment of pathogens and associated infections. Although amphibians undergo regular sloughing of the outer layer of the skin, the potential for regular sloughing to play a role in influencing cutaneous microbial populations and pathogens has been largely overlooked. In the present study, we assessed the effect of skin sloughing on cultivable cutaneous bacterial abundance in the green tree frog (*Litoria caerulea*). We also examined the effects of temperature and hydric environment on sloughing frequency and microbial re-establishment rates. Our data showed that cultivable cutaneous bacterial abundance was significantly reduced by sloughing events, and frogs kept at ‘summer’ temperatures (23–33°C) sloughed almost twice as frequently as those maintained at ‘winter’ temperatures (13–23°C). No effect of hydric environment on sloughing frequency was observed, but we did find that sloughing in *L. caerulea* appeared to be linked to ambient light cycles. Examination of the effect of sloughing on microbial recolonization indicated that at cool temperatures, an extended intermoult interval allowed microbial abundance to reach higher levels than at warmer ‘summer’ temperatures (when the intermoult interval was significantly reduced). Our data suggest that sloughing may significantly influence the establishment and/or maintenance of cutaneous bacterial populations (pathogenic, mutualistic and/or commensal) and this, in turn, may be affected by environmental factors, such as ambient light and temperature. These findings are likely to be important for our understanding of the ecology of skin-based pathogens, such as the amphibian chytrid fungus, *Batrachochytrium dendrobatidis*.

## Abstract (precis)

The skin is the first line of defence in preventing the establishment of pathogens and associated infections in frogs. Regular sloughing of the outer layer can reduce the abundance of cultivable cutaneous microbes in green tree frogs which has ramifications for our understanding of cutaneous pathogens like the amphibian chytrid fungus.

## Introduction

The skin is considered to be a vital component of the immune system because it presents an initial barrier to the establishment of pathogens and associated infections in the body (Proksch *et al.*, 2008; Bos and Luiten, 2009). In vertebrates, the epidermis (the outermost skin layer) is a structurally complex tissue, which undergoes recurrent renewal processes during the lifetime of an organism; these processes ensure that the integrity of the skin barrier is maintained (Dahl, 1993; Bos and Luiten, 2009). Understanding the protective capacity and physiological processes of the skin in relation to varying environmental factors is of interest to current research regarding global environmental change and the emergence of infectious diseases (Harvell *et al.*, 2002; Belden and Harris, 2007; Feder, 2010), because these changes may play a part in the decline of species susceptible to skin-based diseases (Daszak *et al.*, 1999, 2003; Lips *et al.*, 2008; Anchukaitis and Evans, 2010).

In amphibians, the skin serves a variety of essential biological functions, including respiration and osmoregulation, in addition to its role as a physical barrier to infection, making the epidermis a dynamic, permeable surface where many exchanges between the organism and surrounding environment occur (Herman, 1992; Carver *et al.*, 2010). To ensure the continued integrity of the skin surface, vertebrates undergo a skin-renewal process called moulting, or desquamation, whereby the outer stratum of the skin separates from the underlying skin layers and is lost to the environment. Unlike many other vertebrates, in which skin cells are cast off individually or in small patches, amphibians (including anurans, urodeles and caecilians) undergo a more cyclic moulting process whereby an entire stratum of skin is shed each time (Jorgensen and Larsen, 1961; Budtz and Larsen, 1973; Smith, 1975; Herman, 1992; Alibardi, 2003). The amphibian epidermis is composed of a thin, keratinized outer stratum, termed the stratum corneum, immediately below which lies the stratum granulosum, followed by the stratum spinosum, stratum germinativum and stratum basale (Fox, 1994; Guo *et al.*, 2003). During the moulting cycle, the stratum corneum separates from the underlying stratum granulosum, forming a loose, thin layer of skin known as ‘slough’ (Ling, 1972; Smith, 1975). The stratum granulosum then dries and cornifies, becoming the new keratinized stratum corneum, at which time the slough is normally shed from the entire body (Ling, 1972; Smith, 1975). Following a sloughing event, most amphibians will consume their slough (Bendsen, 1956; Herman, 1992; Weldon *et al.*, 1993). The period of time between the recurrent sloughing events is known as the intermoult interval, and this interval can vary from days to weeks depending on the species (Bouwer *et al.*, 1953; Jorgensen and Larsen, 1960, 1961, 1964; Jorgensen *et al.*, 1965; Budtz, 1979; Jørgensen, 1988). The sloughing process is regulated hormonally via the thyroid, adrenal and pituitary glands (Taylor and Ewer, 1956; Jorgensen and Larsen, 1960, 1961, 1964; Jorgensen, *et al.*, 1965; Budtz, 1979; Jørgensen, 1988); however, sloughing frequency and the associated length of the intermoult interval can also be influenced by environmental conditions, including temperature, light and food availability (Bendsen, 1956; Taylor and Ewer, 1956; Stefano and Donoso, 1964; Larsen, 1976; Herman, 1992).

As ectotherms, environmental temperatures can have a major effect on the physiological processes of amphibians. Amphibians also tend to occupy wet, humid habitats and can absorb water cutaneously, although conversely, the permeable nature of their skin makes them very sensitive to dehydration stress (Pounds and Crump, 1994). With a rapidly changing climate predicted to place increasing pressure on amphibian species both as a direct result of temperature on physiological processes but also as a result of changes in relative humidity and rainfall (Bosch *et al.*, 2007; McCaffery and Maxell 2010), it is pertinent and important to examine the effects of environmental variables on the physiology of amphibians.

The typically moist amphibian skin surface provides an ideal medium for the growth of microbes, some of which may be present throughout the life of the organism and some of which are continuously exchanged between the skin and the environment (Culp *et al.*, 2007). These microbes are mostly commensal, and some contribute to the innate immunity of the host amphibian via competitive interactions between species and the production of small antifungal metabolites, such as alkaloids, and larger peptides able to restrict the growth of some pathogens (Lauer *et al.*, 2007; Woodhams *et al.*, 2007; Harris *et al.*, 2009; Becker and Harris, 2010; Lam *et al.*, 2010). Opportunistic microbes that can become pathogenic are also found on the epidermis (Schadich, 2009; Schadich and Cole, 2010; Woodhams *et al.*, 2011). Until recently, very little was known about how the sloughing process and/or sloughing frequency influenced cutaneous microbial abundance. In the cane toad, *Rhinella marina*, we showed that sloughing is thermally sensitive and that sloughing resulted in up to a 100% reduction in cutaneous microbial abundance, suggesting, for the first time, a role for sloughing as a component of the innate immune system of amphibians (Meyer *et al.*, 2012).

**Figure 1:**
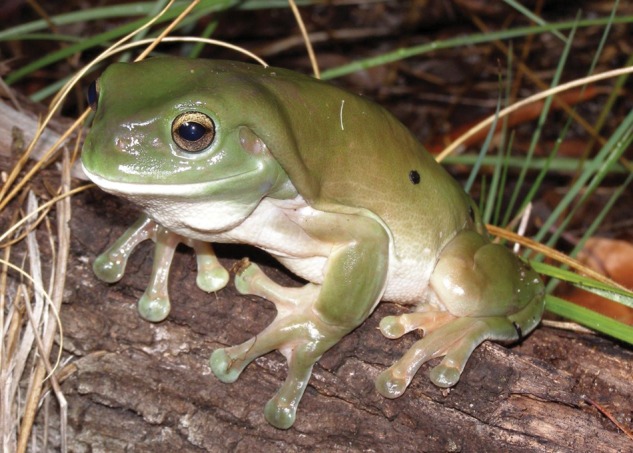
The green tree frog, *Litoria caerulea*. (Photograph by E.A.M.)

Despite the potential significance of these findings in cane toads, questions remain as to whether sloughing in other amphibian species is likewise influenced by temperature or other environmental conditions and if sloughing has the same impact on cultivable microbial communities as in toads. The primary aim of the present study was to investigate the influence of regular sloughing on the cultivable cutaneous microbial abundance of the Australian green tree frog (*Litoria caerulea*; White, 1790; Fig. 1). Green tree frogs were chosen for this study because they inhabit a broad geographical (and thermal) range and have been shown to be highly susceptible to the skin-based pathogen, *Barachochytrium dendrobatidis*. *Litoria caerulea* is also widely used as a ‘model species’ for studies investigating the susceptibility of amphibians to *B. dendrobatidis* (Berger *et al.*, 1998; Voyles *et al.*, 2007, 2009).

In this study, we aimed to assess how thermal and hydric conditions would influence sloughing frequency in order to understand better how climatic and seasonal differences could affect the susceptibility of anuran amphibian species to disease. We hypothesized that sloughing would significantly reduce cutaneous microbial abundance, and that both warmer and drier environments would encourage more frequent sloughing (Taylor and Ewer, 1956; Ling, 1972; Withers, 1995).

## Materials and methods

### Animal collection, husbandry and housing environment

Adult green tree frogs, *L. caerulea* (*n* = 28, mass 23.9 ± 5.73 g, both sexes), were collected from roadsides near Dalby (Queensland, Australia) in March 2011 and transported to laboratory facilities at The University of Queensland. Each animal was housed individually in a ventilated, 5 l plastic container (28 cm × 20 cm × 14 cm), with saturated paper towel on the bottom and a small plastic cup for shelter. The frogs were housed in temperature-controlled cabinets (Percival I-66VLC9; Abacus ALS, Queensland, Australia) set at 23°C on a 12 h–12 h light–dark cycle. Frogs were provided with fresh water and fed with live crickets twice weekly. The paper towel substrate was replaced weekly.

### Experiment 1: effects of temperature on sloughing frequency

To examine the effect of environmental temperature upon sloughing frequency, frogs were marked upon their dorsum with a small smear of non-toxic lipstick (L'Oréal Paris^©^ Colour Riche Intense Lipcolour: Intense Fuchsia). Lipstick has been used in previous studies concerning the regulation of sloughing in amphibians (Bendsen, 1956; Taylor and Ewer, 1956; Budtz and Larsen, 1973; Jørgensen, 1988; Meyer *et al.*, 2012). Lipstick marks were trialled to ensure that they remained on the skin for the entire intermoult period. The loss of a lipstick marker was used to indicate that a sloughing event had occurred.

**Figure 2:**
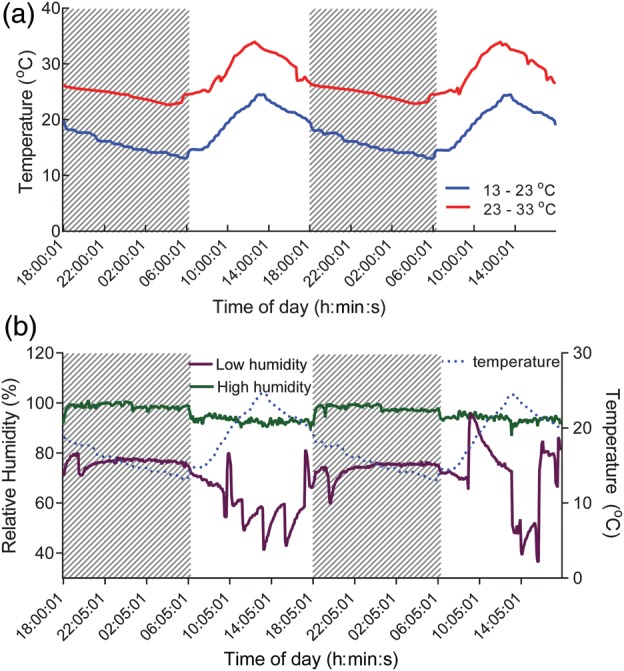
(**a**) Temperature profiles for the controlled temperature cabinets used in the study as represented over 48 h. The 23–33°C regimen is represented by the red line and the 13–23°C regimen by the blue line. Shaded areas represent represent periods of darkness within the cabinet (18.00–06.00 h). (**b**) Humidity profiles for the low relative humidity (purple) and high relative humidity (green) treatment groups within controlled temperature cabinets recorded over a 48 h period. Shaded areas represent periods of darkness within the cabinets. Increased variability of recordings during daylight hours reflect the 2 hourly opening of cabinets to record the sloughing status of frogs.

Immediately after sloughing, frogs were randomly assigned to one of two temperature-controlled cabinets programmed at either 23–33°C (*n* = 14; ‘summer’ treatment) or 13–23°C (*n* = 14; ‘winter’ treatment; Fig. 2). The cabinets were programmed to reflect normal fluctuating temperatures typical of a summer's (23–33°C) or winter's (13–23°C) day in south east Queensland, Australia (Australian Bureau of Meteorology, www.bom.gov.au) and which were within the thermal tolerances of *L. caerulea* (Johnson, 1971; Browne *et al.*, 2007; Pough, 2007; Sammon *et al.*, 2010). Frogs were checked for loss of marks every 2 h between 09.00 and 17.00 h for 33 days. All sloughing events were recorded, and frogs were immediately remarked. The period (in hours) between sloughing events represented the duration of the intermoult interval (IMI). To confirm that the timing of observations was not influencing the calculation of IMI, we then checked frogs every 2 h continuously for an additional 9 days. The duration of the experimental period allowed for the observation of seven to 10 IMIs per animal in the 13–23°C temperature treatment and 12–19 IMIs per animal in the 23–33°C treatment. In each treatment group, we compared the IMI of the initial and final observations, which might indicate the thermal acclimation (compensation) of sloughing. The temperature coefficient (*Q*_10_) value for IMI was calculated using the equation:

$$Q_{10} = \left(\frac{R_{{\rm w}}}{R_{{\rm c}}} \right)^{10/(T_{{\rm w}} - T_{{\rm c}})}$$

where *R* is the mean intermoult interval length for each acclimation group (w, warm group; and c, cold group) and *T* is the mean temperature of each treatment over 24 h (17.0°C for the 13–23°C treatment and 26.8°C for the 23–33°C treatment).

### Experiment 2: effects of hydric environment and humidity on sloughing frequency

As before, frogs were marked on their dorsal surface using lipstick after sloughing had occurred and then randomly assigned to either a dry (*n* = 14) or a wet/more humid hydric environment (*n* = 14). All experiments were conducted at 13–23°C because preliminary trials indicated that low humidity levels in the higher temperature regimen caused frogs to dehydrate too quickly. The low-humidity environment was maintained by placing 100 g silica gel at both the top and the bottom of the constant-temperature cabinet. Animals assigned to this treatment were provided with 10–15 ml water daily (just enough to moisten the paper towel substrate) and were sprayed lightly once daily with water from an atomizer. These conditions maintained a relative humidity within each container of ∼65%. The ‘dry’ environment was representative of conditions that would be experienced in winter in south east Queensland (41–74% relative humidity; Australian Bureau of Meteorology, www.bom.gov.au). The silica gel was replaced daily and reactivated by heating to 120°C for 3 h. The high-humidity environment was maintained by placing two large tubs of water in the temperature cabinet and providing 150 ml of water directly in each animal's container; these conditions resulted in a local relative humidity of ∼95%. Relative humidity was logged continuously in two identical but unoccupied animal containers in each treatment using Hygrochron™ temperature and humidity data loggers (Maxim, Sunnyvale, CA, USA). Animals were checked every 2 h between 09.00 and 19.00 h, from 17.00 h on the first day until 19.00 h on the 14th day and sloughing events recorded. The duration of the monitoring period allowed for the recording of two or three IMIs for each animal.

### Experiment 3: effect of sloughing on cultivable cutaneous bacterial abundance and recolonization rate

**Figure 3:**
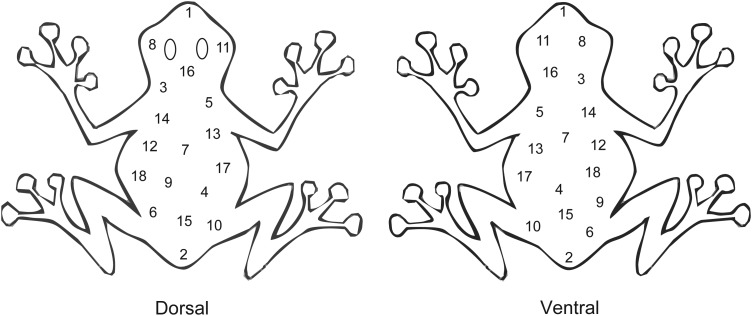
The distinct swabbing locations (1 cm^2^) on the dorsal or ventral skin surfaces of the frogs. Swabbing occurred at one of 18 distinct locations (see numbers 1–18) every 6 h during an intermoult interval, with care taken to swab a different location at each time point.

Frogs (*n* = 6 per temperature treatment) were reassigned to the two thermal regimens as defined above (with high ambient humidity), and the dorsal skin was marked with lipstick. Frogs were maintained in these conditions for 2 weeks prior to experimentation. Prior to handling frogs, all experimental equipment and the bench top were cleaned with 70% ethanol and air dried; this process was repeated between the handling of each frog to prevent cross-contamination between frogs and from the laboratory environment. Frogs were handled with gloved hands, and gloves were replaced between frogs. To assess the rate of bacterial recolonization on the skin during the intermoult interval, frogs were observed until they sloughed. After sloughing, the surface of each frog was blotted lightly with clean paper towel and then immediately swabbed in one dorsal and one ventral locationwith a sterile cotton bud that had been moistened in sterile ultrapure water (Invitrogen, Sydney, NSW, Australia). The cotton bud was rubbed vigorously over ∼1 cm^2^ of the skin surface for 30 s. For the purposes of this study, we did not discriminate between ‘resident’ and ‘transient’ microbes as defined by Lauer *et al.* (2007), but our swabbing procedure would have captured both. The tip of the bud was then cut off and placed into a 1.5 ml microcentrifuge tube containing 1000 μl of sterile ultrapure water. The swabbing regimen was repeated every 6 hours until frogs sloughed again. Care was taken not to swab the same location on the dorsal or ventral surface twice during an IMI by dividing the frog into 18 distinct swabbing sections on each surface and swabbing a different one each time (Fig. 3).

Each microcentrifuge tube containing a cotton bud sample (the primary dilution) was vortexed for 30 s. A 10-fold dilution series (10-, 100- and 1000-fold) was made from the primary dilution and then 50 μl of each dilution plated onto Petrifilm™ nutrient plates for general aerobic bacterial growth [3M Petrifilm Aerobic Count (AC) Plate; Southern Biological, Knoxfield, Victoria, Australia]. Background samples (swabs handled in the same way but with no frog contact) were collected simultaneously and plated on separate plates; negligible contamination was found. Plates were sealed in separate zip-lock bags and incubated at 37°C for 48 h (Heidolph Unimax 1010; John Morris Scientific, Brisbane, Queensland, Australia). Petrifilm™ plates contain a dye, which stains red all bacterial colonies present. Following incubation, the plates were digitized using a flat-bed scanner (Canon CanoScan 8060) and colonies counted using the program SigmaScanPro5 (SyStat Software Inc., San Jose, CA, USA). All colonies were counted, regardless of their size or colour intensity. Colony abundance per square centimetre of skin was then estimated for each surface by multiplying the colony count by the sample dilution factors.

### Statistical analysis

To check the efficiency of the two monitoring protocols used in experiment 1 to record IMI length accurately, we compared the mean IMI length using both protocols and compared these within each temperature treatment using a repeated-measures Wilcoxon signed ranks test. There was no significant effect of monitoring protocol detected (warm acclimated, *W* = 33, *P* = 0.236, 95% confidence interval = −2.34, 3.67; and cool acclimated, *W* = 47.5, *P* = 0.78, 95% confidence interval = −7.43, 13.64); therefore, data were combined for subsequent analyses. To determine whether IMI changed over the experimental period, indicating thermal acclimation, we compared the initial and final IMI using a linear mixed-effects model (*lmer* function in the *lmerTest* package; Kuznetsova *et al.*, 2013). ‘Time’ (initial vs. final IMI length) was regarded as a fixed factor and ‘subject’ as a random effect within the model to control for the autocorrelation of repeated measures from the same subject.

To examine the effects of temperature and humidity on sloughing frequency, the IMIs for each animal were averaged across the experimental period and the treatment groups compared using the Mann–Whitney *U* test.

The efficacy of sloughing in reducing microbial load was calculated by comparing the microbial load on the swab before sloughing with microbial load on the swab immediately after sloughing, initially using a fully factorial linear mixed-effects model (*lmer* within the *lmerTest* package; Kuznetsova *et al.*, 2013). Colony count data were first logarithmically transformed to improve variance and normality. ‘Treatment group’ (warm or cool acclimated), ‘surface’ (dorsal or ventral) and ‘swab number’ (initial or final) were regarded as fixed effects, while ‘subject’ (individual frog) was treated as a random factor. Model simplification was then performed using the *step* function within the *lmerTest* package, which performs backwards elimination on non-significant factors, starting with the random factor. The effects of the random factor were determined using a log-likelihood ratio test and the effects of the fixed factors using ANOVA with *P*-values calculated from *F* statistics of type 3 hypotheses and Satterthwaite approximation for degrees of freedom. Given that subject was insignificant in its effect on colony counts in the *lmer* model (log-likelihood ratio test; χ^2^ = 1.04, df = 1, *P* = 0.307), it was removed, and the data were refitted to a simplified linear model. The effects of the remaining factors on colony counts were determined using ANOVA.

As a result of the difference in the length of the IMI between the two temperature treatments, rates of recolonization between treatments could be compared only over first 48 h post-sloughing, when data from both treatment groups existed. Linear mixed-effects models were fitted to logarithmically transformed colony counts, with ‘treatment’ (warm or cool acclimated), ‘time post-sloughing’ and ‘surface’ (dorsal or ventral) regarded as fixed factors and ‘subject’ as a random factor. Again, model simplification was performed using the *step* function in the *lmerTest* package, with *post hoc* tests of significant differences calculated using the least-squares differences method and confidence interval generated. The effects of the random factor were determined using a log-likelihood ratio test and the effects of the fixed factors using ANOVA of type = 3 with Satterthwaite approximation for degrees of freedom. All analyses were conducted using R (R Core Team, 2013). All data are presented as means ± SEM unless otherwise specified.

## Results

### Effects of temperature and hydric environment on sloughing frequency

**Figure 4:**
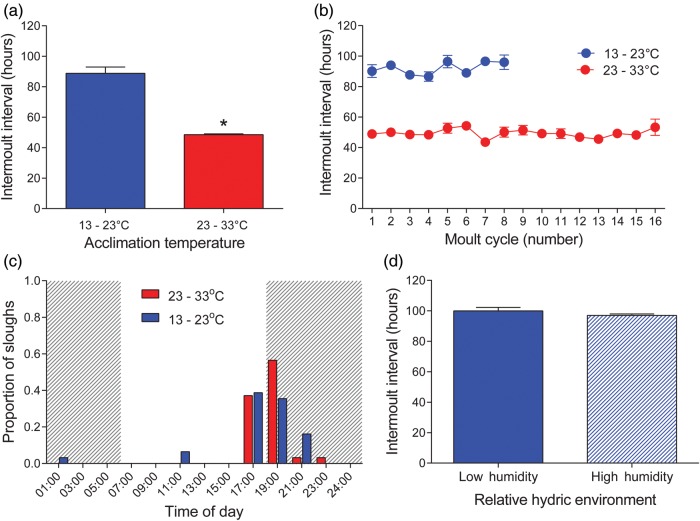
(**a**) The effects of temperature on mean intermoult interval (in hours) in *L. caerulea*. Asterisk indicates that treatment groups were significantly different; error bars represent SEM. Animals within the warmer temperature treatment (23–33°C) showed a significantly shorter intermoult interval compared with those held in the cooler treatment (13–23°C). (**b**) Mean intermoult interval (in hours) for each of the eight moult cycles for frogs at 13–23°C (blue circles, *n* = 14) or 16 moult cycles for frogs at 23–33°C (red circles, *n* = 14). There was no evidence of thermal acclimation in intermoult interval in either treatment. Data are presented as means ± SEM. (**c**) The proportion of sloughing events occurring at each 2 hourly check for each temperature treatment (blue bars, 13–23°C, *n* = 31 sloughing events, from 14 frogs; and red bars, 23–33°C, *n* = 68 sloughing events, from 14 frogs). The majority of sloughing events were recorded at 17.00 and 19.00 h for both treatment groups. (**d**) The effect of hydric environment on mean intermoult interval length (in hours) in *L. caerulea*. Hydric environment had no impact on moult cycle length. Error bars represent +SEM (*n* = 14).

Temperature had a significant effect on the mean length of the intermoult interval (*U* = 0, *P* = 7.2 × 10^−6^, 95% confidence intervals = −47.5, −41.5; Fig. 4a). The mean IMI of frogs in the 13–23°C treatment was 88.8 ± 15.8 h, almost twice that of frogs in the 23–33°C treatment (48.54 ± 1.83 h). The corresponding *Q*10 for sloughing was 1.86. There was no difference in mean IMI length over the 42 days in either temperature group, which indicates that no thermal acclimation (thermal compensation) of sloughing occurred (Table 1 and Fig. 4b). From the 24 h observations of sloughing events, frogs at 13–23°C (*n* = 31 sloughing events, from 14 frogs) were recorded predominantly 1 h before lights went off (17.00 h, 38.7%) and shortly after lights went off at 18.00 h (19.00 h, 35.5%; and 21.00 h, 16.1%). Likewise, frogs at 23–33°C (*n* = 68 sloughing events, from 14 frogs) were recorded to have sloughed mostly between 17.00 h (37.1%) and 19.00 h (56.5%; Fig. 4c).

**Table 1:** Summary statistics for the final model of the effects of prolonged exposure to treatment temperatures on intermoult interval length

Effects	*F* (Dn, Dd)	*P*-value	Confidence intervals	
			2.5%	97.5%
Fixed:				
Time	0.014 (1, 52)	0.906	−9.75	4.60
Temperature	196.8 (1, 52)	<0.001	−52.94	−36.20
Time × Temperature	0.54 (1, 52)	0.467	−5.72	14.58
Random:	χ^2^ (Df)	*P*-value		
Frog	1.89 (1)	0.169		

Dn, numerator degrees of freedom; Dd, denominator degrees of freedom; Df, degrees of freedom.

Sloughing cycles were recorded for 42 days in 28 green tree frogs (*Litoria caerulea*) and the lengths of the initial and final cycle were compared using a linear mixed-effects model (*lmer* function in *lmerTest* package in R) to determine whether any acclimation occurred over the experimental period. There was no change in mean intermoult interval over the period of measurement.

There was no significant effect of hydric environment (at 13–23°C) on mean IMI length over the experimental period (*U* = 74.5, *P* = 0.288, 95% confidence intervals = −6.667, 2.333; Fig. 4d), with the mean IMI for the animals in the dry and wet treatments being 99.9 ± 8.7 and 96.9 ± 3.7 h, respectively. There was no change in IMI over the monitoring period in either treatment group (Table 2).

**Table 2:** Summary statistics for the final model of the effects of prolonged exposure to wet and dry hydric conditions on intermoult interval length

Effects	*F* (Dn, Dd)	*P*-value	Confidence intervals	
			2.5%	97.5%
Fixed:				
Time	1.38 (1, 26)	0.246	−2.53	9.67
Humidity level	0.56 (1, 51)	0.455	−7.57	5.57
Time × humidity level	0.11 (1, 26)	0.742	−10.20	7.06
Random:	χ^2^ (Df)	*P*-value		
Frog	0.5 (1)	0.48		

Dn, numerator degrees of freedom; Dd, denominator degrees of freedom; Df, degrees of freedom.

Sloughing cycles were recorded for 14 days in 28 green tree frogs (*Litoria caerulea*) and the lengths of the initial and final cycle compared using a linear mixed-effects model to determine whether any acclimation occurred over the experimental period. There was no detectable change in mean intermoult interval length over the measurement period.

### Effect of sloughing on cultivable microbial abundance and post-slough recolonization rate

**Figure 5:**
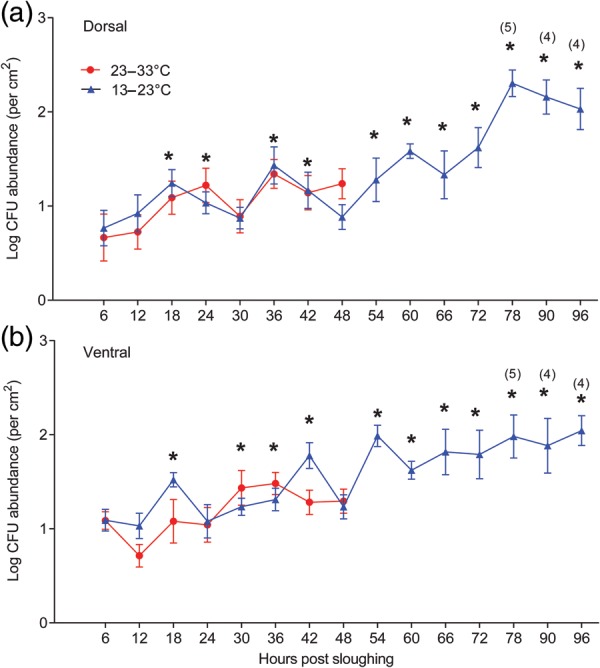
Recolonization trends of cultivable bacterial abundance (log bacterial colonies per square centimetre) recorded every 6 h over the intermoult interval of frogs kept at 13–23°C (blue triangles, *n* = 6) and 23–33°C (red circles, *n* = 6) for the dorsal (**a**) and ventral skin surfaces (**b**). Microbial abundance increased significantly over the first 48 h following sloughing. There was no effect of treatment temperature on microbial abundance in the first 48 h, but microbial loads were greater on the ventral surface during this period. The difference in colony-forming unit (CFU) abundance between the first and last slough was significantly different between temperature treatments, with animals in the cooler treatment having a significantly greater microbial abundance prior to the final slough relative to animals in the warm treatment. Numbers in parentheses represent the number of animals at that time point which had not yet sloughed. Asterisks indicate a significant difference from the CFU abundance at time = 6 h post-sloughing. Error bars show means ± SEM.

General aerobic bacteria were cultured from both dorsal and ventral body surfaces. Within the samples collected from each skin surface and within temperature treatments, there was considerable variation in bacterial numbers prior to sloughing. Bacterial abundance on both the dorsal and ventral skin surfaces increased significantly during the intermoult period (*P* < 0.0001), but the effect was more pronounced in the cold-temperature treatment group (*P* = 0.0011; Table 3 and Fig. 5). There was no difference in colony-forming unit (CFU) abundance across the two skin surfaces (*P* = 0.12). During the intermoult period, microbial abundance in the warm treatment increased by ∼3.8- and ∼4.6-fold (ventral and dorsal surfaces, respectively), while in the cold treatment, microbial abundance increased by 25- and 32-fold (ventral and dorsal surfaces, respectively). The rate of increase in cultivable micro-organisms was relatively low in both temperature treatments in the first 48 h following sloughing (Fig. 5). Although treatment temperature had no overall effect (*F*_(1,_ _10)_, *P* = 0.69; Table 4), there were strong effects of time post-sloughing (*F*_(7,_ _157.98)_ = 8.53, *P* = 1 × 10^−7^), skin surface (*F*_(1, 157.98)_ = 16.92, *P* = 1 × 10^−4^) and interactions between time post-sloughing and temperature treatment (*F*_(7,_ _157.98)_ = 2.18, *P* = 0.038) and time post-sloughing and skin surface (*F*_(7,_ _157.98)_ = 2.14, *P* = 0.042) on CFU abundance during this period.

**Table 3:** Summary statistics for the final model of the effects of treatment temperature on the efficacy of sloughing in reducing bacterial load on the skin surfaces (dorsal and ventral)

Effects	*F* (Dn, Dd)	*P*-value	Confidence intervals	
			2.5%	97.5%
Fixed:				
Temperature	12.24 (1, 42)	0.001	−0.56	0.81
Swab number	49.48 (1, 42)	<0.001	1.96	3.13
Surface	2.46 (1, 42)	0.124	−0.08	0.76
Temperature × swab	17.32 (1, 42)	<0.001	−2.74	−1.06
Random:	χ^2^ (Df)	*P*-value		
Frog	1.04 (1)	0.31		

Dn, numerator degrees of freedom; Dd, denominator degrees of freedom; Df, degrees of freedom.

Treatment temperature, swab number (initial or final) and skin surface (dorsal or ventral) were included in the fully factorial model as fixed effects, while subject was considered a random effect to account for autocorrelation of repeated measures on the same animal. Model simplification and *post hoc* analyses were conducted using the *step* function in the *lmerTest* package in R.

**Table 4:** Summary statistics for the final model of the effects of temperature and skin surface on the change in colony-forming unit abundance over time (recolonization) of dorsal and ventral skin surfaces

Effects	*F* (Dn, Dd)	*P*-value
Fixed:		
Temperature	0.17 (1, 10)	0.69
Time	8.53 (7, 158)	<0.001
Surface	16.9 (1, 158)	<0.001
Temperature × time	2.18 (7, 158)	0.038
Time × surface	2.14 (7, 158)	0.042
Random:	χ^2^ (Df)	*P*-value
Frog	38.86 (1)	<0.001

Dn, numerator degrees of freedom; Dd, denominator degrees of freedom; Df, degrees of freedom.

Data were initially fitted to a fully factorial linear mixed-effects model, with treatment temperature, time post-sloughing (in hours) and skin surface as fixed factors and subject as a random factor. Model simplification and *post hoc* analyses were conducted using the *step* function in the *lmerTest* package in R.

## Discussion

Sloughing frequency and the length of the associated IMI are significantly influenced by temperature but not hydric environment in *L. caerulea*, and the timing of sloughing events appears to be sensitive to ambient light levels. In *L. caerulea*, sloughing also had a significant impact on cultivable cutaneous microbial abundance, with microbial numbers on both the dorsal and ventral surfaces being markedly reduced after sloughing events. The efficacy of sloughing in the abundance of microbes on the skin's surface suggests that sloughing could serve as one of the first lines of defence against the establishment and subsequent incursion of pathogenic microbes at the body's surface.

Results from our study suggest that sloughing frequency can be influenced by temperature. As in *L. caerulea*, sloughing frequency has been shown to increase with increasing temperatures in several other anuran species, including *Bufo bufo* (Bendsen, 1956), *Amietophrynus (Bufo) regularis* (Taylor and Ewer, 1956) and *R. marina* (Meyer *et al.*, 2012). In *B. bufo*, a small change in temperature range from 16–18 to 20–23°C resulted a decrease in mean IMI of almost 4 days, with IMI dropping from ∼13 to 9 days. Likewise, in *Amietophrynus regularis* (Taylor and Ewer, 1956) and *R. marina* (Meyer *et al.*, 2012), animals kept at ∼30°C sloughed twice as frequently as those at ∼20°C.

The difference in sloughing frequency regimen between the experimental temperature treatments in this and previous studies raises questions about the influence of season on sloughing periodicity. To address this question, we also investigated whether sloughing frequency is subject to thermal acclimation or not (by looking for changes in IMI over an extended period of 42 days). The length of the IMI of *L. caerulea* in this experiment was unaffected by exposure to treatment temperatures for 42 days, suggesting that sloughing may not be a process that is subject to thermal acclimation. This finding is consistent with the findings of Meyer *et al.* (2012), who also found no evidence for thermal acclimation of sloughing frequency in *R. marina*. The fact that relatively low temperatures reduce sloughing frequency in all amphibian species studied to date raises the possibility that microbes could have greater success at establishing themselves upon the epidermis of amphibian hosts at high latitudes, where microbe numbers are possibly less strongly regulated by frequent host sloughing regimens.

The IMI for *L. caerulea* was significantly less than that reported for bufonids, in which sloughing frequency ranges from 3 to 14 days (between 15 and 30°C ambient temperature; Bendsen, 1956; Taylor and Ewer, 1956; Meyer *et al.*, 2012). Sloughing of *L. caerulea* was, however, not as frequent as recorded in some desert-dwelling, cocoon-forming frogs, where sloughing can occur as frequently as every other day (Withers, 1995) or even daily, as seen in leaf-frogs of the genus *Phyllomedusa* (Castanho and De Luca, 2001). However, it remains unclear exactly how much variability in skin shedding frequency occurs in amphibians and whether there is any correlation between intrinsic skin sloughing frequency and the development of disease from cutaneous pathogens.

Unlike temperature, the hydric environment did not significantly affect sloughing frequency in *L. caerulea*. This finding contrasts with other studies that have shown that amphibians in drier environments tend to slough more frequently than those in more humid environments (Ling, 1972; Withers, 1995). To ensure the viability of the stratum corneum within a dry environment with low relative humidity, it is thought that a desiccation stress response triggers more frequent sloughing so that mucus produced as a part of the sloughing process can keep the epidermis moist (Larsen, 1976; Bustamante *et al.*, 2010). However, this does not appear to be the case in *L. caerulea*, which did not respond to desiccation or dehydration by increasing sloughing frequency. Whether this is because animals were not sufficiently stressed or because the hypothesized link between sloughing frequency and hydric conditions is species specific remains unclear and is a topic for future work.

In both temperature treatments, dorsal and ventral cultivable cutaneous bacterial loads were significantly reduced by sloughing events, by as much as 100% in some cases. The marked reduction in bacterial abundance after sloughing in *L. caeruela* is consistent with our previous findings in *R. marina* (Meyer *et al.*, 2012). Analysis of cutaneous bacterial recolonization rates following sloughing also showed that animals at lower temperatures harboured a greater number of culturable bacterial colonies per unit of skin surface at the end of the IMI relative to animals in the warmer treatment. The relative brevity of the IMI in frogs in the warmer treatment is therefore likely to have limited the extent to which cultivable bacteria on the skin surface could multiply before sloughing. Conversely, the greater IMI of frogs in the cooler treatment permitted cutaneous microbial numbers to increase substantially before sloughing occurred.

The results of this study and that of Meyer *et al.* (2012) suggest that sloughing frequency plays a significant role in the regulation or management of cutaneous microbial abundance on frog skin. In both studies, bacteria cultivable using our approach are likely to have represented only a small portion of the total microbial flora present on the skin surface (Amann *et al.*, 1995). Nevertheless, there is little reason to doubt that sloughing would have the same or a similar impact on non-cultivable microbes inhabiting the outer skin surface, because sloughing in amphibians normally involves the complete separation and removal of the outermost layer of skin cells and all that is adherent to that layer. How quickly microbial communities are restored following sloughing is likely to be both microbial species specific and temperature dependent. The higher bacterial loads found upon the skin prior to sloughing at the end of a long IMI are not simply dependent upon the thermal biology of the host amphibian and its response via sloughing frequency, but are also dependent on the thermal biology and competitive ability of the microbes present (Piotrowski *et al.*, 2004).

Although there was no difference in the efficacy of sloughing in reducing microbial loads on both skin surfaces in *L. caerulea*, data from the recolonization experiment suggest that there were different rates of microbial accumulation between the two skin surfaces, with the ventral surface having a somewhat greater load of bacteria relative to the dorsal surface. The distribution of microbes, including pathogens and commensal bacteria, has been found by some researchers to vary greatly both in relation to the different amphibian skin surfaces and also with regard to specific location upon the skin surface (e.g. Berger *et al.*, 2005; Becker *et al.*, 2009). The ventral surface of most amphibians is in almost constant contact with the environment, which facilitates the rapid transfer of micro-organisms, while the dorsal surface has a greater distribution of antimicrobial peptide-producing glands (Goniakowska-Witalińska and Kubiczek, 1998), so microbial loads are often lower. Culp *et al.* (2007) postulated that microbes recolonize skin surfaces largely through direct environmental contact but also suggested that some bacteria may reside in skin glands, because they noted an accumulation of microbes around some gland openings in a small number of individuals. Consistent with this, Loudon *et al.* (2013) have recently demonstrated that the immediate environment greatly influences the microbial diversity of the ventral surface of red-backed salamanders (*Plethodon cinereus*). Animals maintained on a natural soil medium maintained a high microbial diversity over time relative to animals maintained in relatively clean conditions. Despite the differences in environment, salamanders also appeared to maintain a ‘core community’ of microbial species, several of which have known antifungal activity (Loudon *et al.*, 2013). The source of microbes repopulating the skin of *L. caeruela* after sloughing is unknown but is likely to include the environment. Whether *L. caerulea* also maintained a ‘core community’ of bacteria and, if so, the source of these also requires further investigation.

The findings of this study have implications for research into amphibian cutaneous microbes and disease, especially *B. dendrobatidis*, and the response of such emerging infectious diseases and amphibian hosts to environmental change (Bendsen, 1956; Taylor and Ewer, 1956). *Batrachochytrium dendrobatidis* is found within the superficial epidermal strata, the stratum corneum and the stratum granulosum (Van Rooij *et al.*, 2012), and is tolerant of a wide range of temperature and precipitation conditions, with growth rates maximized in cooler, temperate thermal regimens both *in vitro* and in the field (Johnson *et al.*, 2003; Woodhams *et al.*, 2003; Piotrowski *et al.*, 2004; Berger, *et al.*, 2005; Chatfield and Richards-Zawacki, 2011; Murphy *et al.*, 2011). *Batrachochytrium dendrobatidis* spores are also transmitted via water; therefore, the hydric environment or water availability is also considered important to the establishment and severity of infection (Kriger and Hero, 2007; Woodhams *et al.*, 2008).

In previous studies, amphibians transferred to relatively high temperatures (30–37°C) have been reported successfully to clear *B. dendrobatidis* infections previously contracted at cooler temperatures, possibly through a direct effect of temperature on the fungus (Woodhams *et al.*, 2003; Chatfield and Richards-Zawacki, 2011) but also through the impacts of temperature on host sloughing frequency. Although these studies did not examine the rate of sloughing, the findings from the present study indicate that exposure to such a high temperature could have increased sloughing rates, which in turn may have contributed to clearance of *B. dendrobatidis* infections. It is possible that increased sloughing by frogs kept at high temperatures means that *B. dendrobatidis* can be lost from the skin before it has a chance to complete its life-cycle. Consistent with this view, sloughing has even been recorded as a factor in false-negative diagnosis of chytridiomycosis in animals where testing for *B. dendrobatidis* has occurred soon after a sloughing event (Olsen *et al.*, 2004). Thus, by regulating the build-up and establishment of *B. dendrobatidis* in keratinized skin, sloughing could significantly affect the susceptibility of frogs to *B. dendrobatidis* infection.

Whether sloughing plays a role in determining the susceptibility of amphibians to *B. dendrobatidis* remains relatively unexplored. Anecdotally, *B. dendrobatidis*-infected amphibians are reported to shed more frequently than non-infected animals (e.g. Voyles *et al.*, 2011); however, the reliability of this observation has been questioned (Meyer *et al.*, 2012) because diseased amphibians are often inappetent, and the appearance of an increased abundance of skin sloughs in the enclosures of sick frogs may simply reflect their reluctance to consume their shed slough as healthy amphibians do routinely. Nevertheless, given that mature zoosporangia are found in the stratum corneum (Van Rooij *et al.*, 2012), sloughing of this layer and the presence of uneaten slough might help to spread the infective stages of the parasite if sloughing is co-ordinated with the maturation cycle of *B. dendrobatidis*.

In conclusion, our data show that sloughing frequency of *L. caerulea* varies according to temperature, with frogs kept a warmer temperatures sloughing more frequently than those kept at cooler temperatures. Also, the burden of cultivable cutaneous bacteria is significantly reduced by sloughing and then increases again over the course of the intermoult interval. Taken together, our data suggest that for frogs at cooler temperatures, the longer intermoult interval means there is a greater opportunity for cutaneous microbes to accumulate between sloughing events. As such, more regular sloughing may help to limit the build-up of pathogenic microbes at the body's surface, thereby helping to protect frogs from infection. However, as the skin also harbours communities of beneficial microbes, which exclude or inhibit pathogenic microbes, frequent sloughing could potentially increase the susceptibility of animals to infection by rapidly reproducing pathogens. This may mean that frogs that have a high intrinsic sloughing frequency could even be at greater risk of disease than frogs that slough less frequently. Whether sloughing frequency lessens or increases the risk of disease from pathogenic organisms, such as *B. dendrobatidis*, is unclear. A greater understanding of the variation in sloughing frequency amongst amphibians is required to provide a better understanding of the epidemiology of skin-borne diseases, such as *B. dendrobatidis*. Future work also needs to examine more closely the relation between normal sloughing and cutaneous microbes like *B. dendrobatidis*, as well as temporal changes in cutaneous microbial populations with sloughing.
